# Pediculosis

**Published:** 1910-01-01

**Authors:** 


### Dermatology.

### PEDICULOSIS,

The human species has the privilege of providing sustenance for no less than three distinct sorts of pediculi, known according to their respective habitations as P. capitis, P. vestimentorum, and P. pubis. No one of these varieties is conspicuous by reason of its rarity, and pediculosis capitis is doubtless the commonest of all skin affections. To its well-nigh universal prevalence among the children of the lower classes County Council nurses can testify. Few mothers regard the presence of nits in the head of their offspring as a reproach, although they will admit that the scalp should be kept free from the mobile forms of insect life. When a child in whom pediculosis capitis is well established is brought to the hospital for treatment the usual complaint is that it has lumps in its neck. Judicious inquiry will elicit the clinical history of lumps in the neck followed by a breaking out in the head, and subsequently by the appearance of " things " ascribed by the mother to a disordered state of the blood. This, of course, is an exact reversal of the true sequence of events, which is the lodgment of a gravid pediculus or a pair of nits. the breeding of numerous descendants, consequent irritation and scratching, leading to the development of impetigo contagiosa with the enlargement of the lymphatic glands of the posterior triangle of the neck.

It is a curious and somewhat mysterious fact that each variety of louse should confine its activities to its own sphere of influence with religious exactitude, whether one sort alone be present or, as not uncommonly happens, all three share the same individual. This must be considered the very culmination of specialism. The only possible exception to this rule is found possibly in the case of pediculosis of the eyelids, for the pediculus palpebrarum is said to be identical with the pediculus pubis. Everyone, of course, is now aware that the only means of contracting pediculosis is by infection, but not so many years since it was held by high medical authorities that some unfortunate individuals had the peculiar property of breeding pediculi in their unbroken skin; they were said to suffer from the " lousy diathesis," more politely called phtheiriasis, a word derived from the ancient Greek name of the insect. A considerable body of literature has collected on the subject during many years, but the doctrine of phtheiriasis only received its *coup-de-grace* from the researches of Hebra about 1866. During the Middle Ages, and far more recently than that, it was unquestioned, and countless quotations might be made in illustration of it from the most respected and learned authors.

The three species of pediculi with which we are now concerned resemble one another in many ways. Unlike the organism associated with scabies, the Sarcoptes or Acarus, which is a biological relation of the spider tribe, they are true insects, though of a low order, for they do not undergo any metamorphosis in the course of their development as the higher insects, such as the bees and butterflies, do. The P. vestimentorum is the largest of the three, but has a narrower thorax than the P. capitis, while the P. pubis is the broadest of the three and possesses no clear demarcation between the thorax and abdomen. The most interesting feature in the structure of the pediculi from our point of view is the anatomy of the blood-sucking organs. These consist of a proboscis, which is a prolongation of the œsophagus, armed with four palpi to select a suitable spot for operations, and a pair of mandibles with which to bite the skin. Their life history is uneventful, but their power of multiplication is terrific. A single louse may lay fifty eggs within six days. These take from three to eight days to hatch, and are themselves capable of laying within three weeks. Within eight weeks, therefore, a single louse may be the ancestor of some 5,000 descendants. As regards the symptoms due to the presence of these pests, the severe eczema and impetigo so often observed are entirely secondary, and caused by the scratching and rubbing employed to allay the distressing irritation. The primary lesion of pediculosis is merely a tiny red speck which can be seen but not felt. It is found on examination with the microscope that the spot chosen by the pediculus is always the orifice of a sweat-gland. Whether the scratching and rubbing of the unfortunate patient causes mere excoriation, or pus formation and the production of abscesses, depends on cleanliness and the general conditon. In children pustulation is the rule.

Of the three forms P. vestimentorum is the easiest treated. All that is necessary is application of a parasiticide ointment and the disinfection of the polluted garments on which the body-louse carries out all its life-processes except feeding, and on which its ova are deposited. The lice which infest the hairy parts require more prolonged treatment, because the ova are attached to the hairs by a tubular sheath which is secreted round the hair by the parent and prevents the ovum from being easily dislodged or picked off. The only way a nit can be removed from its hair is by sliding it down to the distal end of the hair between the finger and thumb. This, of course, is too laborious when one reflects on the vast number of nits there may be on a badly infected head. A more practical method is the application of a small toothcomb after treatment of the head with a mixture of spirit and acetic acid, which kills the adults and softens the chitinous egg-cases. The regular use of these measures will soon cure the dirtiest head. The irritation caused by the P. pubis is not so severe as that produced by the other pediculi, but is more prolonged. Any itching in the region liable to be infected should give rise to a suspicion of the presence of the pediculus. The cleanliest persons are liable to be infected, for although the ova are deposited near the bases of the hairs, the adult louse often balances itself quite insecurely near the distal end, and is liable to be knocked off. Hence public conveniences are a fruitful source of infection. The application of a mercurial ointment is a specific remedy.

